# Can the Psycho-Emotional State be Optimized by Regular Use of Positive Imagery?, Psychological and Electroencephalographic Study of Self-Guided Training

**DOI:** 10.3389/fnhum.2016.00664

**Published:** 2017-01-12

**Authors:** Svetla Velikova, Haldor Sjaaheim, Bente Nordtug

**Affiliations:** ^1^Smartbrain ASOslo, Norway; ^2^Faculty of Nursing and Health Science, Nord UniversityBodø, Norway

**Keywords:** imagery, mood, EEG, LORETA, connectivity, coherence, emotions, self-guided

## Abstract

The guided imagery training is considered as an effective method and therefore widely used in modern cognitive psychotherapy, while less is known about the effectiveness of self-guided. The present study investigated the effects of regular use of self-guided positive imagery, applying both subjective (assessment of the psycho-emotional state) and objective (electroencephalographic, EEG) approaches to research. Thirty healthy subjects participated in the cognitive imagery-training program for 12 weeks. The schedule began with group training with an instructor for 2 days, where the participants learned various techniques of positive imagery, after which they continued their individual training at home. Psychological and EEG evaluations were applied at the baseline and at the end of the training period. The impact of training on the psycho-emotional states of the participants was evaluated through: *Center for epidemiologic studies- Depression (CES-D) 20 item scale, Satisfaction with life scale (SWLS) and General Self-Efficacy scale (GSE)*. EEGs (19-channels) were recorded at rest with eyes closed. EEG analysis was performed using Low resolution electromagnetic tomography (LORETA) software that allows the comparison of current source density (CSD) and functional connectivity (lagged phase and coherence) in the default mode network before and after a workout. Initial assessment with *CES-D* indicated that 22 participants had subthreshold depression. After the training participants had less prominent depressive symptoms *(CES-D, p* = *0.002)*, were more satisfied with their lives *(SWLS, p* = *0.036)*, and also evaluated themselves as more effective *(GSE, p* = *0.0002)*. LORETA source analysis revealed an increase in the CSD in the right mPFC (Brodmann area 10) for beta-2 band after training (*p* = *0.038)*. LORETA connectivity analysis demonstrated an increase in lagged coherence between temporal gyruses of both hemispheres in the delta band, as well as between the Posterior cingulate cortex and right BA21 in the theta band after a workout. Since mPFC is involved in emotional regulation, functional changes in this region can be seen in line with the results of psychological tests and their objective validation. A possible activation of GAMK-ergic system is discussed. Self-guided positive imagery (after instructions) can be helpful for emotional selfregulation in healthy subjects and has the potential to be useful in subthreshold depression.

## Introduction

Emotion-centered imagery training is seen as a powerful tool and is widely used in modern cognitive psychotherapy. But almost all the knowledge achieved during its six decades of history is associated with guided imagery, meanwhile research exploring the efficacy of self-guided is still very limited. Estimation of the potential of self-guided imagery, for example- if the regular practice of imagery containing a positive scenario, created in accordance with current needs can have a beneficial effect on the emotional condition—is a question of practical importance. If the answer is positive, this might offer new strategies to combat negative emotions in our daily lives. Here we investigated the impact of self-guided positive imagery training on emotions and brain functions.

As originally stated in the Lang's bio-informational theory of emotional imagery (Lang, 1977, 1979), mental imagery differs from verbal thought in that only mental imagery has the capacity to activate physiological and behavioral response system (Lang, 1987). It has been shown significant heart rate acceleration, relative to baseline during mental imagery, but not during verbal repetition of fearful scenarios (Vrana et al., 1986; Cuthbert et al., 2003), suggesting the imagery has easier access to our emotional system than the information provided by other sources, such as verbal (Holmes and Mathews, 2005). Thus, imagery can easily provoke an emotional disturbance. On the other hand, all mental disorders are accompanied by any dysfunction in image generation, such as: A deficit or excess in generation of images, an imbalance in the generation of positive and negative images, image distortion. A classical example of a severe violation in the imagery system is the post-traumatic stress disorder, where the presence of post-traumatic flashbacks is considered hallmark of the disorder (Ehlers et al., 2002). The potential of imagery systems is less effective when one is concerned, rather than when there is positive thinking (Hirsch et al., 2012). This deficiency is more pronounced in people with generalized anxiety (GAD) than in a control group (Hirsch et al., 2012). According to a study of Morina et al. (2011), the presence of anxiety was associated with a higher ability to generate bright images for potential negative scenarios and a deterioration of the ability to generate positive images (also true for depressive individuals) compared with control group. Likewise, as shown by Holmes et al. (2008), the imagery abilities were also related to the different personality traits, such as high dysphoria (compared to low) was associated with worsening ability to vividly imagine positive but not negative future events. On the other hand, distortions in imagery may be related to the facts of the past and can affect interpersonal relationships through altered judgments about past events (Garry et al., 1996; Sharman et al., 2004). In brief, the negative emotions and disturbances in the imagery system form a vicious circle thus supporting the fact of emotional imbalance.

Guided cognitive imagery interventions demonstrated the ability to break this cycle, and therefore are widely used in modern cognitive psychotherapy in the treatment of phobias, anxiety, depression, etc. (Wolpe, 1958; Anderson and Borkovec, 1980; Hackmann et al., 2011; Ji et al., 2016). Furthermore, the emotion-centered imagery training have demonstrated the ability to increase optimism (Blackwell et al., 2013), to improve interpersonal relationships (Meleady et al., 2013), as well as in the promotion of empathy and pro-social behavior (Gaesser, 2012) in healthy individuals. Although there is a vast knowledge about the beneficial impact of guided emotion-centered imagery on the emotional life, the effectiveness of self-guided training has yet to be explored. *One* of the obstacles for these types of assessments is to have only limited information (Huang et al., 2014) in relation to “how” emotional imagery affects the brain, as this information may allow an objective assessment of the impact of training. Therefore, here we explored the effect of self-guided emotion-centered imagery, trying to answer not only the question “whether”, according to the subjects, there was an effect on their state of emotional well-being, but also “how” functioning of the brain was affected by training. Objective effects on brain function were tested comparing the electroencephalograms (EEGs) recorded at rest with eyes closed, collected at baseline and after 12 weeks of practicing self-guided emotion-centered positive imagery training. The analysis of EEG obtained in this state can provide information about the functioning of the structures included in the default mode network (DMN) and the functional connectivity of the network, which are reported to be modified in the course of various mind-body practices (Brewer et al., 2011; Berkovich-Ohana et al., 2014; Garrison et al., 2015). The analysis of EEG resting state and the resting state networks was implemented through the use of standardized and exact low resolution brain electromagnetic tomography (sLORETA, eLORETA) (Pascual-Marqui, 2002, 2007a; Pascual-Marqui et al., 2011). LORETA neuroimaging software was used previously by other authors for the study of emotions (Saletu et al., 2010), in addition, this approach has been applied to investigate DMN in relation to practice different mind-body techniques (Lehmann et al., 2012; Berkovich-Ohana et al., 2014).

Here we hypothesized, that if efficiently, then self-guided positive emotion-focused imagery training will demonstrate a beneficial effect on the emotional state of the trainees called out by psychological testing (first hypothesis), and will be linked with EEG changes, explainable by the training (second hypothesis), namely: (a) from a topographical point of view, it could be expected changes in the structures/regions participating in the imagery or/and emotional processing; (b) anticipated increase in EEG connectivity [in accordance with previously reported data on the practice of visual imagery (Sviderskaya et al., 2006)]; (c) since the imagery training by itself is a creative work, some of the changes in EEG are supposed to occur in theta range, which is one of the most frequently reported as involved in the creative process (Petsche, 1996).

## Materials and methods

### Subjects

Participants in the study were thirty volunteers (24 women and six men) aged 20–55 years (mean age 35.5 years). They were collected through an announcement in the social media (Facebook). Initially, semi-structured interviews with a psychiatrist were conducted to assess the psycho-emotional state and motivation of candidates. Excluding criteria were psychosis and affective disorders according ICD-10 (ICD-10, World Health Organization, 1993). Candidates with subthreshold depression were included. All the participants had “normal” life and social activity. They were free of medications and/or other medical interventions. The participants had no previous experience in mindfulness or meditation techniques and didn't have other types of mental training or psychotherapy during the observed period. Before the start of the study an informed consent from all participants was obtained. This study was conducted in accordance with the Declaration of Helsinki^1^.

### Training program

**Figure 1:**
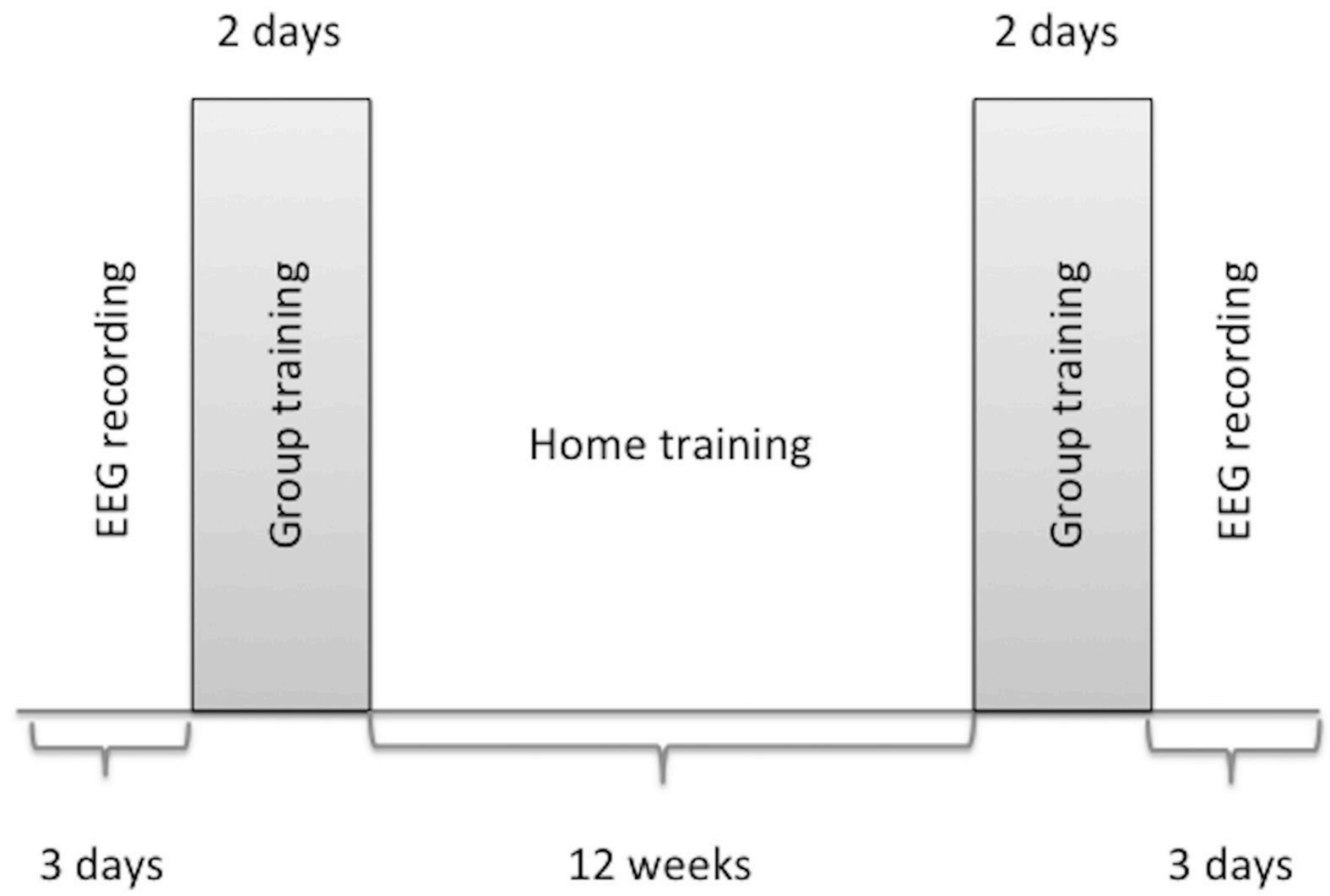
**Design of the study**.

The program duration was 12 weeks and included an initial 2-day lasting seminar with guided group training, followed by practice at home (15–20 min/daily) and then a *second* group training (2-day lasting) at the end of the observed period (Figure 1). The participants learned techniques in order to use imagery:

– To cope with the past psycho-traumatic events (through imagery transformation of a psycho-traumatic event to positive one);

This exercise was based on the imagination of the circumstances before the psycho-traumatic event and requires visualization of alternative positive story. Participants were asked to work with the important event every day for many days, until the moment when they are easy to “see” the story with a positive ending.

– For the goal achievement (through positive imagery of future events);

For achievement goals of a high level of significance for the person, the participants were instructed to *first* describe as detailed as possible goals and steps to achieve it. The *second* stage was to visualize the steps and ultimate goal as if they already been achieved (recommended repetition of the exercises).

– To improve the social interactions (through positive imagery of social interactions and imagery the emotions of other people);

In cases with important interpersonal conflicts in the past, which have a negative impact on current emotional life, the participants were asked to “restore mental” conflict situation visualizing friendships. For future events based on social interactions, the participants learned to imagine the future atmosphere of tranquility, openness and a positive result in link to these event. The participants were encouraged to experience empathy when visualize the scenes.

– To enhance the emotional balance in daily life (participant learned to visualize the next day in a positive way);

When visualizing “tomorrow” the focus was on the mental representation of calmness and freshness, while awakening, and peace and satisfaction at the end of the day.

In addition, the participants were instructed to write a self-report on the regularity of the work performed. The sessions at home began with relaxation during countdown from 7 to 1, followed by imagery exercises thematically adapted to the current needs.

### Psychological assessment

Before the start of the experiment and after the end, participants were asked to perform a self-evaluation as follows:

- In order to determine the depression quotient, *the Center for epidemiologic studies depression (CES-D) 20 item scale* (Radloff, 1977) was used. CES-D is a self-report depression scale for research in the general population and measures the depressive feelings and behavior during the past week.
- *Meaning in life questionnaire (MLQ)* (Steger et al., 2006; Steger and Shin, 2010), designed to measure two dimensions of the meaning of life: (1) the presence of meaning (the extent to which participants feel that their lives have meanings), and (2) search for meaning (the extent to which respondents strive to find meaning and understanding in their lives).
- *Satisfaction with life scale (SWLS)* (Diener et al., 1985), developed as a measure of the judgmental component of subjective well-being.
- *General Self-Efficacy scale (GSE)* (Schwarzer and Jerusalem, 1995). The scale reflects the presence of optimistic self-confidence (Schwarzer, 1992), that is, the belief that one can perform a novelty or difficult task, or to cope with adversity in various domains.

A paired-sample *t*-test was performed (SPSS.13) to compare the values of psychological tests obtained before and after accomplishment of the training.

### Electroencephalographic (EEG) recording and analysis

The EEG recording and analysis of the data were conducted at Smartbrain AS Oslo. Electroencephalograms were obtained at baseline (up to 3 days prior to the start of the training), and after accomplishment of the training (up to 3 days after the end of the observed period of 12 weeks) in resting state with eyes closed for 5 min.

Nineteen-channel EEGs were recorded with a Discovery amplifier (BrainMaster, USA^2^) using ElectroCap [electrodes were positioned according to the International 10/20 system (Jasper, 1958)]; linked ears were used as reference. The impedance of the EEG signal was below 5 Ω; the sampling rate was 256 Hz. These EEG data were visually inspected and artifacts were removed manually using NeuroGuide Deluxe (Applied Neuroscience Inc., Florida, USA) software version 2.8.3 (AppliedNeuroscienceInc)^3^. From each EEG-recording was selected artifact-free segment with a length of at least 60 s, having test-retest reliability coefficient higher than 0.95.

The edited EEGs records were further analyzed using LORETA software (Pascual-Marqui, 2002; KEY Institute for Brain-Mind Research, Zurich)^4^ version 20150415. LORETA resolves the inversion problem, maximizing the power of synchronization only between neighbor neuronal populations and allows 3-D reconstruction of electrical density of electrical sources of EEG signal. The solution space of LORETA is defined via a reference brain from the Brain Imaging Center at the Montreal Neurological Institute (MNI). Computations were made in a realistic head model using for inverse solution the electric potential lead field computed with boundary element method (Fuchs et al., 2002), applied to the MNI152 template (Mazziotta et al., 2001). The cortical surface used by LORETA is based on Van Essen average cortex (Van Essen, 2005). The software reports MNI coordinates (Jurcak et al., 2007). Anatomical labels as Brodmann areas are reported using an appropriate correction from MNI to Talairach space (Brett et al., 2002). Therefore, LORETA images represent the electric activity at each voxel in neuroanatomic Talairach space (Talairach and Tournoux, 1988) as the squared standardized magnitude of the estimated current density.

For comparison of the electroencephalograms obtained before and after training, two types of analysis using LORETA software were conducted: The functional localization and functional connectivity.

#### Functional localization

Analysis of the functional localization was implemented using sLORETA software. This is a method for estimating the localization of cortical generators, performing source localization in 6239 cortical gray matter voxels sized 5 mm^3^. Initially, the CSD in the EEG recorded at baseline and after exercise was assessed for each person. Then, comparison the distribution of CSD between the paired groups was implemented using non-parametric statistical analysis (Statistical non-Parametric Mapping; SnPM) employing the Log of ratio of averages (log of F-ratio) and performing SnPM randomization (number of randomizations = 5000). The SnPM methodology corrects for multiple comparisons and does not require gaussianity assumptions (Nichols and Holmes, 2002).

#### Functional connectivity

Functional dynamic connectivity in the brain is used to be quantified by coherence and phase synchronization, but these measures are highly contaminated with an instantaneous non-physiological contribution due to volume conduction and low spatial resolution (Pascual-Marqui, 2007b). Provided by eLORETA coherence and phase analysis removes this confounding factor considerably, solving the problem by decomposing these parameters into instantaneous and lagged components, where the latter have almost pure physiological origin (Pascual-Marqui, 2007b). Therefore, here we chose to investigate lagged linear connectivity (coherence) and lagged non-linear connectivity (phase synchronization). For analysis of the connectivity at the outset seven regions of interests (ROI) were preselected [in correspondence with the areas belonging to the default mode network (DMN) (Raichle, 2011)]:

- Left Medial Frontal Gyrus (BA9-L)
- Right Medial Frontal Gyrus (BA9-R)
- Posterior Cingulate (BA31L+BA31-R)
- Left Inferior Temporal (BA-21L)
- Right Inferior Temporal (BA21-R)
- Left Lateral Parietal (BA39-L)
- Right Lateral Parietal (BA39-R)

In order to compare functional connectivity between the selected regions before and after the training, t-statistic for paired groups, followed by SnPM randomization (*n* = 5000) was carried out.

Both functional localization and functional connectivity analyses used the next frequency bands: Delta (1.5–4 Hz), Theta (4.5–8 Hz), Alpha1(8.5–10 Hz), Alpha2 (10.5–12 Hz), Beta1 (12.5–18 Hz), Beta2 (18.5–21 Hz) and Beta3 (21.5–30 Hz).

## Results

### Psychological testing

Initial assessment with CES-D showed that 22 participants had subthreshold depression (score higher than 16, which represents cutoff for “non-significant” or “mild” depressive symptomatology (Radloff, 1977), but they did not meet the ICD-10 criteria for depression). At the end of the training, according to CES-D, participants had less prominent depressive symptomatology (M = 16.83, *SD* = 5.73), than at baseline (M = 19.93, *SD* = 7.54); *t*_(29)_ = −3.355, *p* = 0.002, and the number of them having score more than 16 was reduced to 12 (non of them met the criteria for depression). The subjective assessment regarding the “presence of meaning in life” after training was higher (M = 25.43, *SD* = 4.42) than before the training (M = 23.63, *SD* = 5.53); *t*_(29)_ = 2.335, *p* = 0.027. At the same time, there was no change in the category “search for meaning in life” after training (M = 25.77, *SD* = 5.5) from baseline (M = 25.67, *SD* = 6.24); *t*_(29)_ = 0.120 (*p* = 0.906). As shown by “Satisfaction with life” questionnaire, after the training the participants were more satisfied with their lives (M = 20.77, *SD* = 6.11) than in the past (M = 18.93, *SD* = 4.32); *t*_(29)_ = 2.20, *p* = 0.036. In accordance with “General Self-Efficacy scale”at the end of the training, they also perceived themselves like more effective (M = 33.03, *SD* = 4.58) than before the training (M = 30.67, *SD* = 4.15); *t*_(29)_ = 4.372, *p* = 0.0002.

### EEG results

**Figure 2:**
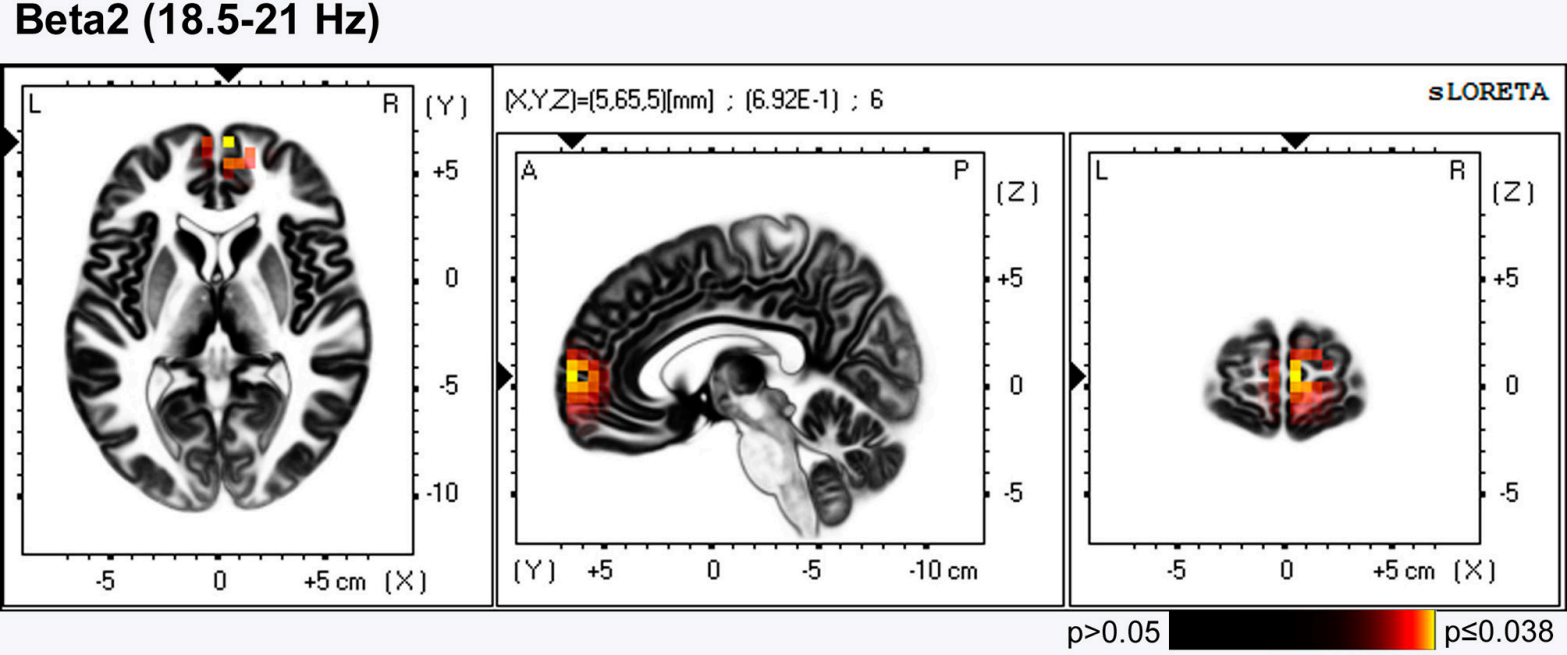
**LORETA group comparison of the current source density(CSD) before and after training, employing Log of the F-ratio statistic, and SnPM randomization**. The voxels with increase in CSD in the Right medial prefrontal cortex- Brodmann area 10 [beta2 band(18.5–21 Hz)] are presented in red (0.038 ≤ *p* ≤ 0.05) till yellow (*p* = 0.038).

sLORETA comparison demonstrated that after the training there was significant (*p* = 0.038) increase in the current source density (CSD) in the Right medial prefrontal cortex (mPFC)- Brodmann area 10 for beta-2 band (18.5–21 Hz) (Figure 2).

**Figure 3:**
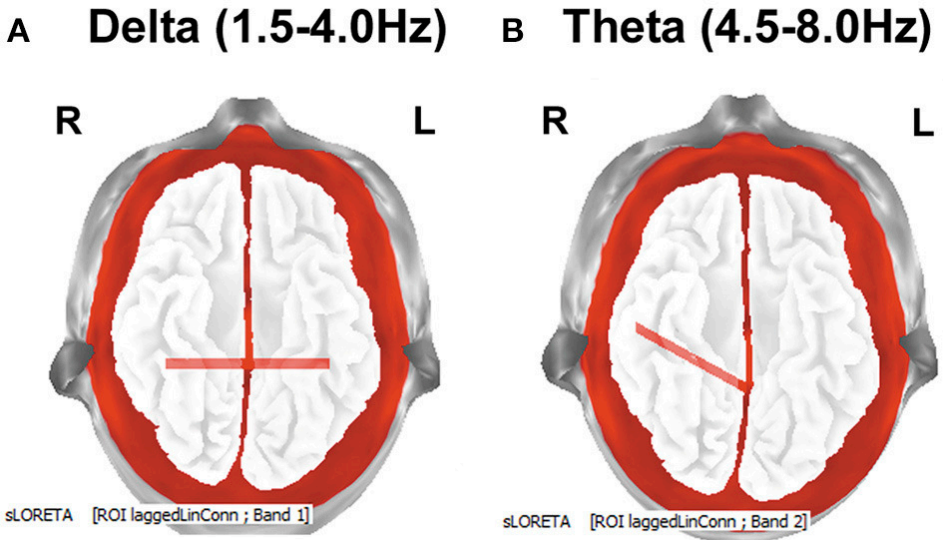
**LORETA group comparison of lagged coherence before and after training, employing *t*-statistic, and SnPM randomization**. The distances with significantly increased after the training lagged coherence are indicated with red lines. The results are presented according to the EEG bands: **(A)** delta (1.5–4 Hz): increased lagged coherence between Inferior temporal gyruses from both hemispheres; *p* = 0.035. Bottom view. **(B)** theta (4.5–8 Hz): increased lagged coherence (*p* = 0.04) between posterior cingulate cortex (PCC) and right Inferior temporal gyrus (BA21). Bottom view.

Connectivity analysis (eLORETA) between the selected ROIs showed after the training increased lagged coherence between the Inferior temporal gyruses (BA21) from both hemispheres in delta band (*p* = 0.035), as well as between the posterior cingulate cortex (PCC) and right Inferior temporal gyrus (BA21) for theta band (*p* = 0.04) (Figure 3). There were no significant changes in the lagged phase.

EEG parameters showing significant differences were subjected to correlation analysis with the data from psychological testing, but no correlation was observed.

## Discussion

After 12 weeks of positive imagery training, the participants perceived themselves in a more positive light and had a feel for more meaning in their lives. In the end, they were more satisfied with their own lives, which confirmed our *first* hypothesis. The observed dynamic in the emotional state of the trainees, as pointed out in previous studies on the guided mental imagery, proved, that the methods of eliminating troublesome images and creating healthy alternatives (Hackmann and Holmes, 2004), as well as techniques to boost the positive image of the future (Blackwell et al., 2013) have a beneficial impact on the emotional state. Furthermore, the present study found that a positive emotional transformation may also be obtained, when the visualization is a self-guided exercise and thematically adapted to the current needs.

The analysis of EEG data confirmed the *second* hypothesis; it was validated that the regular practice of positive imagery cause changes in brain oscillatory activity and there are some arguments to indicate that the EEG changes can be seen in reference to the training. Firstly, the 3-D topographic analysis using sLORETA showed an increase in the current source density (CSD) in the right mPFC (BA10) after the training. Given that this region is involved in imagination of pleasant scenes (Costa et al., 2010), and also plays a role in self-reflection and self-awareness (Johnson et al., 2002), the observed changes in this area can be seen as being related to the training program. The fact of increased CSD in BA10 after training from other point of view is in line with the results of psychological tests, since this region is involved in the regulation of emotion (Liotti et al., 2002), as well as, contributes for the degree of satisfaction with life (Kong et al., 2015), parameters that were significantly changed after training. Our findings are consistent with data from meta-analytic reviews of neuroimaging studies in healthy participants, according to which mPFC is *one* of the structures, along with the anterior cingulate cortex, amigdala (Phan et al., 2002; Murphy et al., 2003) and the Insular cortex (Craig, 2009) most consistently associated with emotional processing.

Secondly, lagged coherence analysis demonstrated increased (as originally hypothesized) value of EEG coherence after a workout. *One* of the observations was increased inter-hemispheric coherence between the inferior temporal gyruses of both hemispheres. As these regions that are involved in the processing of images (Mellet et al., 1996), enhancing the coherence between them may indicate that the training caused an improvement of coordination of relevant networks located in temporal gyruses. Increased EEG coherence has been reported in connection with the visual imagery of simple graphical elements (topographic distribution and bands of observed changes have been different, depending on previous experience) (Sviderskaya et al., 2006), so it can be assumed that increased inter-hemispheric lagged coherence between inferior temporal gyruses is associated with the image forming process in general, but do not reflect the content of the images. Further, it can be supposed that the increase in the functional connectivity between the PCC and the right inferior temporal gyrus is associated with recurrent self-image processing in the course of the training, as among the various functions, PCC also plays a role in the self-referential process (Garrison et al., 2015). Moreover, self-referential process has been previously associated with a group of theta frequencies (Mu and Han, 2010), the same frequency range, where the observed changes described here.

Another hypothesis to consider in connection with the observed changes in the EEG, is the possible contribution of enhancement activity of GABA (gamma-aminobutyric acid) -ergić system, which is well-known for its anti-anxiety and antidepressant properties (Möhler, 2012). The *first* argument in favor of this hypothesis due to the fact that LORETA changes were found in beta range, which appears to be modulated by GABAergic system (Yoto et al., 2012). At the same time, it has been shown that GABA enhancement leads to increased interhemispheric EEG coherence (Fingelkurts et al., 2004; Sampaio et al., 2007). Thus, the possible contribution of GABA (gain at the end of training, compared to baseline) can be seen in agreement with the results of both analyzes. Indeed, it has been shown that practice of mind-body techniques, such as yoga, can lead to enhancement of GABA (Streeter et al., 2007).

One can argue about the need for the control group, performing only relaxation (in order to distinguish the effect of relaxation and imagery training) or other image script, for example, to play football (in order to distinguish the effect of the imagery scenario). In regard to this, we believe that the relaxation itself can't explain the observed results, not only because of the time constraints of relaxing exercises, but also because the results in this study are consistent with those observed during the imagery and contrast changes seen in other studies throughout the relaxation: EEG studies on relaxation techniques reported in the main decrease in coherence in the DMN (Lehmann et al., 2012; Berkovich-Ohana et al., 2014), which is opposite of the observed here and in other studies dedicated on imagery in which increased coherence was demonstrated (Sviderskaya et al., 2006). With regard to the option “control group, imaging different scenario,” we believe that such an option is incorrect, because it gives equal task to all of the participants. Consequently this makes the training of guided instead of self-guided, so unsuitable for further comparison because *one* and the same scenario can have different emotional content for the participants, which breaks down the main principle that the images must have a positive content for the person.

The knowledge gained from this study show that the self-guided imagery training (after adequate guided training) may be useful as a part of self-development programs to improve the emotional well-being in healthy subjects, and has the potential to be cost-effective method of intervention for subthreshold depression.

## Ethics statement

The project was reviewed and approved by Regional Ethics Committee South East Norway (D 2016/921).

## Author contributions

SV analyzed the EEG data and wrote the article. HS recorded EEG, participated in the discussion and interpretation of data. BN analyzed the data from psychological tests and participated in the discussion and interpretation of data.

## Funding

This study was partially supported by the Nord University, Norway.

### Conflict of interest statement

The authors declare that the research was conducted in the absence of any commercial or financial relationships that could be construed as a potential conflict of interest.

## Footnotes

^1^WMA Declaration of Helsinki-Ethical principles for medical research involving human subjects [Online]. (Accessed 1964).

^2^Brainmaster Technologies Inc., B., Oh, USA. Available online at: http://www.brainmaster.com

^3^http://www.appliedneuroscience.com [Online]. USA.

^4^The Key institute for brain-mind research. Zurich: University Hospital of Psychiatry. Available online at: http://www.uzh.ch/keyinst/
